# Methods for Evaluating Pozzolanic Reactivity in Calcined Clays: A Review

**DOI:** 10.3390/ma16134778

**Published:** 2023-07-02

**Authors:** Valber Domingos Pinheiro, Jonas Alexandre, Gustavo de Castro Xavier, Markssuel Teixeira Marvila, Sergio Neves Monteiro, Afonso Rangel Garcez de Azevedo

**Affiliations:** 1 Laboratory of Advanced Materials, State University of the Northern Rio de Janeiro, Campos dos Goytacazes 28013-602, Brazil; valber.pinheiro@hotmail.com; 2 Civil Engineering Laboratory, State University of the Northern Rio de Janeiro, Campos dos Goytacazes 28013-602, Brazil; jonasuenf@gmail.com (J.A.); gxavier@uenf.br (G.d.C.X.); 3 CRP-Rio Paranaíba Campus, UFV-Federal University of Viçosa, Rio Paranaíba 38810-000, Brazil; markssuel.marvila@ufv.br; 4 Department of Materials Science, IME—Military Institute of Engineering, Square General Tibúrcio 80, Rio de Janeiro 22290-270, Brazil; snevesmonteiro@gmail.com

**Keywords:** methods, pozzolanicity, clay, reactivity, calcined

## Abstract

The search for alternative materials to replace ordinary Portland cement has been the object of work that enhances the investigation of the use of pozzolanic materials and the reduction of the carbon footprint with supplementary cementitious materials. However, not all materials are available to meet the large-scale demand for cement replacement. A relevant exception is the calcined clay, a material found worldwide that, when subjected to appropriate heat treatment, presents pozzolanic reactivity and can be used as a supplementary material to cement. This review presents, through a systematic search, methods for measuring the pozzolanic reactivity of calcined clays, namely, direct, indirect, qualitative, quantitative, chemical and physical methods such as electrical conductivity (Lùxan), the force activity index, the modified Chapelle, R^3^, Frattini test, thermal analysis, X-ray diffraction and X-ray fluorescence spectrometry. The most usual methods to assess the pozzolanic reactivity of calcined clays were exposed and analyzed. It should be pointed out that there is greater use of the Frattini and modified Chapelle methods as well as the analysis of the mechanical strength behavior of the material in cementitious matrices. X-ray diffraction and thermal analysis were exposed as the most used correlation methods but it was also concluded that different tests are needed to generate accurate results.

## 1. Introduction

The use of ordinary Portland cement (OPC) has a long history of applications and has been considered reliable in building construction and versatile since its application can be performed in a wide-range of environmental conditions and work contexts [1]. However, the production of OPC requires a considerably higher energy expenditure if compared with the production of some types of supplementary cementitious materials (SMCs) such as fly ash, slag and calcined clays for example. One can also highlight that the production of OPC has a greater environmental impact if one takes into account both the extraction of raw materials that are not renewable and the significant amount of pollutant gases that are dumped directly into the ecosystem.

The partial replacement of OPC by SCM can reduce CO_2_ emissions by up to 40% without significant interference in important characteristics of the cementitious matrices such as strength and durability properties. The benefits of using SCMs in cementitious matrices include increasing long-term strength, reducing permeability and decreasing shrinkage reactions that might cause micro-cracks in already hardened concrete structures [2].

Likewise, the use of SCM contributes to reducing the energy expenditure of production and consequently, generates a lower manufacturing cost [3] considering that OPC is still used as a material on a large scale [4]. In summary, this type of supplementary replacement allows a reduction of the environmental impact of cement production without harming the ease of use and versatility of cement. These characteristics can try to make this type of supplementary material with significant worldwide use. Moreover, most SCMs are siliceous and reduce the heat of hydration of some cementitious systems, providing inherent favorable characteristics in certain applications. Most of the available cements in many countries are already dosed with SCMs, such as fly ash, slag and limestone [1]. Additionally, non-calcinated clays [5], vegetable ashes, such as those from sugar cane bagasse, elephant grass and rice husk [6], limestone filler [7], marble rock and clay filter residue [8], natural clays and calcine clays [1,9,10], bamboo leaf ash [11], granite quarry waste [12], as well as metakaolin [13,14,15], volcanic rocks [16], are SCM and also considered pozzolans. However, it must be considered that there is a limitation in the use of most of these products due to their relative scarcity or location that hinders their extraction and increases costs associated with the transfer to the processing plants. Avet et al. [13] point out that the mixture of OPC clinker with SCM in increasing levels is a feasible alternative for obtaining more sustainable cements. On the other hand, despite their high potential properties in cement matrices, some of these supplementary additions when used together with OPC may not meet its growth production over the years. As a consequence, besides materials such as fly ash and blast furnace slag that have limited production, there is a great interest in studying the calcined clays for use in this purpose. Thus, among the SCM, the calcined clay is pointed out as a potential candidate to fill this gap since this material has been widely studied in different parts of the world [17]. The manufacture of OPC clinker, burning limestone with clay, requires a temperature between 1200 and 1500 °C which results in high CO_2_ emission due to the decomposition of raw materials [18]. The calcination of clays, on the other hand, is performed at a considerably lower temperature, between 450 and 900 °C, not presenting expressive CO_2_ release. It is important to emphasize that the clays used must be predominantly kaolinitic. The purpose of calcination is the transformation of kaolinite into metakaolinite, which occurs between 450 and 900 °C. Thus, it is possible to observe that crude clay minerals with low chemical reactivity, in general, are not applied as a direct use without an activation treatment, such as thermal activation by calcination, for example [19,20]. The calcination at higher temperatures can cause the transformation of metakaolin into mullite. However, the calcination of clays should be carried out at a controlled temperature, which is lower than the clinker production temperature. Calcination is a heat treatment that can consist of a temperature ramp-up, plateau or residence time, and cooling method. This process is considered endothermic and is used on materials such as clays. Besides changing the crystalline structure, it has the ability to remove volatile elements from the samples, oxidize organic matter if calcined in air, eliminate unwanted impurities, improve electrical conductivity, produce important oxides and perform thermal decomposition for the activation of different clays evaluating the reactivity and measuring the pozzolanic activity of the calcined material [21]. During the calcination process of clays, thermal transformations are observed, for both the argillominerals in isolation and the clay minerals as a whole. However, there is a need for better studies and correlations about the optimal temperature and duration of calcination since divergent information is still presented in the literature [22]. This divergence is mainly due to the variety of clay minerals and components, often inert, that make up the clay material in its entirety. Thus, it is essential to adjust the particular and exact firing conditions for each individual type of clay, whether kaolinitic, illite, montmorillonite or the others. It is worth mentioning that besides clays, there are other materials that when undergoing activation processes enable the formation of materials with a high reactivity index such as myrene residues. Materials such as calcined clays are defined as SCMs and are used in cementitious matrices with the purpose of enhancing properties and as a sustainable solution in the partial replacement of OPC [23]. SCMs can also improve the workability of concrete and provide cost savings by replacing the more expensive OPC [24,25]. The use of calcined clays is further leveraged by the seasonal and regional factor in which a large portion of the pozzolanic materials studied do not have wide availability of raw materials and do not keep up with the cement industry in projection [26]. The use of calcined clays as supplementary materials to OPC influences the filling effect, the reaction speed due to nucleation and pozzolanic reactivity [27]. Currently, clays are mainly used as an indispensable raw material in the production of artifacts in the ceramics industry, such as bricks, and as a main element in the raw mass of the cement industry [28]. To analyze the pozzolanic potential of the SCM, tests and characterizations, either direct or indirect, help in the quantification and qualification of pozzolanic reactivity particularizing the inert materials from the reactive ones. For scientific productions that cover research with pozzolanic materials, the most commonly used methods for measuring the pozzolanicity index, according to [29], are the Frattini test, modified Chapelle, determination of pozzolanic activity through electrical conductivity, fast, reproducible and reliable or R^3^, colorimetry, X-ray diffractometry (XRD) and thermogravimetric analysis (TGA) pointing out correlations between tests and methods. The tests for the evaluation of pozzolanic activity are divided into direct and indirect methods [30]. The direct methods are those that measure the consumptions of calcium hydroxide remaining from the hydration reaction of OPC, also known as portlandite (CH), by the pozzolanic reaction. These direct methods correlate the changes in chemical reactions and temperatures by means of chemical titration, XRD and thermal gravimetric analysis (TGA). The indirect methods, which measure a property change, also known as mechanical methods, evaluate the pozzolanic reactivity of SCMs by measuring the mechanical performance, strength activity indices, mechanical strength, electrical conductivity and heat conduction [31].

The objective of this review is to compile and discuss the most used and effective methods for evaluating the pozzolanicity of clays, especially calcined clays, addressing their advantages and disadvantages as well as the correlations between the different methods. In this context, a comparison of different methodological approaches will be presented for the characterization of parameters that affect the pozzolanic reactivity and for the evaluation of the reactivity of the supplementary material after calcination. The specific objectives were developed with (i) further discussion of direct and indirect methods of evaluating the pozzolanicity of supplementary materials; (ii) understanding of the particularities related to pozzolanic reactivity tests for clays; (iii) observation of the mechanisms of development for reactivity analysis; and (iv) discussion regarding the importance of concomitant analysis of the results from different methods with the purpose of a better accuracy of the reactivity of calcined clays. Furthermore, the in-depth study of this research work will contribute to advancement on the knowledge of reactivity of supplementary cementitious materials.

## 2. Sources of SCM and Pozzolanic Materials

SCMs are composed of fine particles that have similar or complementary characteristics to those of the OPC. This type of material is generally used in replacement or addition to cement, allowing an improvement of performance in the fresh and hardened state of cementitious matrices that can compose part of the OPC in a supplementary way with hydraulic or pozzolanic properties. Significant shortages and demand are still observed in the recognition of chemical and physical correlations such as hydration, hardening time, mechanical strength, porosity, shrinkage and fineness of cementitious mixture formulations, thus seeking a sustainable and efficient application of the processing of reuse materials, discarded materials and already consolidated materials, for different applications in cementitious matrices in order to improve or grant properties [32]. Table 1 demonstrates the similar or complementary characteristics of OPC with those of SCM [23].

The use of supplementary cementitious materials as partial substitutes for conventional OPC or binder materials such as hydrated lime [8], is an alternative for reducing CO_2_ emissions, considering that concrete, even nowadays, is an indispensable component of civil construction [31]. As an alternative, there remains the development of products and materials that can make concrete from OPC an environmentally sustainable option [33]. However, the main criterion for a material to be a substitute or complementary for OPC is the contribution provided by them to the improvement of mechanical properties [34], and the formation of the products similar to those of OPC hydration when in contact with calcium hydroxide, in considerable fineness, alkaline aqueous medium and suitable conditions. The addition of SCM in OPC systems influences the hydration reaction through physical effects and chemical reactions [35]. When added to a cementitious system, an SCM tends to guarantee properties in the fresh and hardened state, presenting the physical effects that occur during the reaction constituted by the presence of fine and inert powders, which improve properties through the filling effect and a better particle size [36]. Chemical reactions participate in the formation of hydration products with the mechanism of dissolution and precipitation involving the solid reactants, the reaction products of the solid hydrates and the solution. In the case of SCM use, the chemical reactions can be pozzolanic, when C-H is consumed during the process, or hydraulic. The benefits of its use include increased long-term strength, decay of porosity, reduced permeability and reduced shrinkage reactions that can cause microcracking in already hardened concrete structures [2].

Observing the studies of Adu-Amankwah [37] and Skibsted and Snellings [3], it is possible to infer that the use of materials rich in aluminum and silica require a high amount of water in the system for the formation of the compounds in order to obtain greater reactivity in their formation. The effects of the particles of pozzolanic materials (siliceous and aluminum minerals) when added to the cement mixture containing water, react with the Ca(OH)_2_ and form an alkaline medium that enables the production of silica with a mineralogical breakdown of the clay. As a result, a greater pozzolanic reactivity and production of secondary C-S-H, also known as tobermorite, are obtained [38,39]. With this, it is possible to observe a considerable variation in reactivity which reflects in the final hydrated phase, microstructure, cement performance, higher strengths and durability due, in large part, to prolonged hydration [3].

The reactivity of SCM is given by several factors that go beyond the raw composition of the material. Among these factors, one can consider the activation of the material, its degree of fineness, the hydration vehicle aqueous medium and the previous thermal treatment. There are the ones that most interfere in the degree of reactivity of each of the materials. When it comes to the reactivity of these materials, the pozzolanic activity that these materials hold not only needs to be addressed, but also the physical and chemical effects that can interfere with the performance of the material altogether [3]. However, most SCMs when combined with OPC still do not allow a complete hydration reaction even if the availability of time is large. This limitation is strictly related to the availability of reactants such as Ca(OH)_2_ and water that are consumed during the process and, due to this, it becomes impossible to conduct the reaction in high levels of hydration. The non-availability of water in the system or its total consumption may cause a reduction in the internal relative humidity of the concrete and cause resistance loss, shrinkage and increased porosity in the cement matrix, that are unwanted or, in some cases, detrimental. Similarly, the particle size, directly linked to the refinement process of these materials, is one of the main factors that promotes greater durability to the cementitious matrix. Thus, during hydration, the initial porosity is filled by the reaction products, and as the process goes on, the spaces are occupied, making a greater expansion and growth of hydrates impossible [3]. According to Avet et al. [13], OPC mixtures with higher substitution contents of calcined clay or metakaolin are more prone to this process, in turn forming more products of the aluminate family. The fineness of SCM is highlighted as a main physical property that can affect performance. A material that has a high degree of fineness for example can provide the cementitious matrix with both advantages and disadvantages. The dissolution of this fineness causes an increase in their surface area, thus enabling an acceleration of hydration at early ages with the filling effect. The effect of particle size refinement results in a more homogeneous interface between the particles of the cementitious matrix making mortars and concretes, for example, stronger. In addition, the decrease in particle size causes an increase in the hydration of the system and again, better performance results in properties such as strength are achieved [3]. Similarly, the chemical parameters also present a great amount of interference in the reactivity of the SCM. The reactive components in SCM generally occur in the glassy phases, formed by silicates and aluminates, or amorphous. Their reactivity is determined by their composition and chemical structure [3]. The reactivity of these materials is developed from the dissolution capacity that is modified according to the rate of heating, cooling and concentration of the solution at different hydrogen potentials (pH). CH is an element largely responsible for regulating the pH of cementitious matrices such as concrete and mortar in order to reduce the corrosion and degradation vehicle of steel reinforcement or elements present in these compounds [40]. However, many materials used as a supplement to cement have the characteristic of altering the alkalinity of the medium, generating future concern with the structures in which these materials are applied [3,38,39]. However, the ability to dissolve by chemical processes in SCM is often due to alkali–silica reactions, geopolimerization and alkaline activations that have as a product a gel with moisture absorption capacity, i.e., hygroscopic characteristics [41,42]. These elements in general tend to expand after absorption of free water causing an increase in volume causing small cracks and thus leading to a decrease in durability [38,39]. Ideally, a cement substitute material to be used in cementitious matrices such as concrete and mortar should have a good pozzolanic reactivity and with devices that can mitigate the expansion caused by alkali–silica reactions and exaggerated transport of chloride ions that are enhanced by the fineness of the material [43].

Pozzolans are natural or artificial material, in general, containing the phases of silicate and aluminate which tend to react with the C-H remaining in the hydration reaction of OPC producing C-S-H [44,45,46,47]. With this, there is a demand for research that involves different materials with pozzolanic potential. The ashes of vegetable origin, for example, are pozzolans studied with the purpose of developing more sustainable cementitious elements and with enhanced properties. The use of rice husk ash [48,49,50] and sugar cane bagasse ash [51] promotes the characteristics of better durability and increased mechanical strength when used in mortars and concretes behind lower shrinkage rates and decreases in the emission of pollutant gases such as CO_2_. Other materials are used with this same purpose. Similarly, fly ash as an SCM is used as a potential addition to cementitious matrices in order to enable new properties and improve the performance of mortars and concretes [52,53,54,55]. As with other types of pozzolans, clays also occupy a place in replacing cement in mortars and concretes [56]. When used in the natural form, there are concerns about the degree of porosity that clay can provide in mortars and other pathologies such as carbonation. As for the calcined ones, the biggest concern is their pozzolanic reactivity, i.e., the consumption of C-H that clays will have within a paste hydration system. However, a limiting factor in the pozzolanic reactivity of calcined clays is the distinction between clay sources that present quantitative and qualitative divergences in their argillominerals. However, these materials give, as a result, an element rich in silica, alumina and other minerals in smaller quantities. The calcined clay is pointed out as a material with potential in improving the performance of mortars and concretes that provides the cementitious matrices with higher strength and lower porosity due to the filling effect and pozzolanic reactivity that this material holds [57]. Indeed, the clays are subjected to a calcination-type heat treatment in order to provide properties such as lower shrinkage by drying, decreased water absorption and electrical conductivity, decreasing permeability and the already expected increased mechanical strength. Above all, calcined clays present similar characteristics to other cement substitute materials, and as a result, comparative analyses are carried out in order to investigate the particularities of each one. From the different analyses of the production of cements mixed with different materials, it can be observed that the use of calcined clay does not always represent an improvement in common characteristics such as consistency and workability. This is due to the dehydroxylation of the material that generates higher water consumption after calcination [58,59]. As a source of calcined clays and in order to reduce the carbon footprint, one of the major objectives of using SCM, the reuse of materials or use of waste from clay materials that have previously undergone some activation process is performed. Consequently, such material is used to compose the cementitious matrices with the objective of obtaining greater pozzolanic activity, mechanical behavior similar to mortars and concrete references or improvement in mechanical strength [60,61].

## 3. Analysis of Pozzolanic Activity

The pozzolanic reactivity of SCM measures the degree of reaction over time between a pozzolan and C-H in the presence of water [62]. The properties of cementitious matrices containing pozzolanic material come from a direct acid–base reaction, called pozzolanic, which uses calcium hydroxide and free silica to form C-S-H. This procedure can take an extensive amount of time to be completely developed.

**Figure 1:**
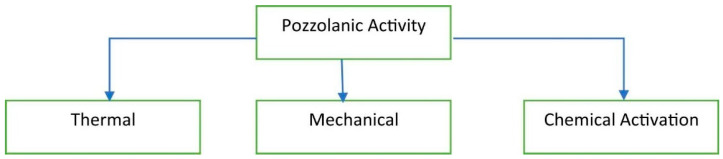
Pozzolanic reactivity [62].

There are different methods for analyzing the pozzolanic reactivity of substitute or complementary materials to OPC, which can be subdivided into three large groups according to Figure 1. There groups are (i) thermal analysis; (ii) mechanical analysis and (iii) chemical analysis; not to mention the characterization methods that, early on, indicate pozzolanic activity. Generally, the verification of the pozzolanic reactivity of the materials must be carried out in a partitioned manner. First, chemical, petrographic and mineralogical analyses are performed, and then, in a second phase, methods or tests that verify the reactivity of the material are arranged.

**Figure 2:**
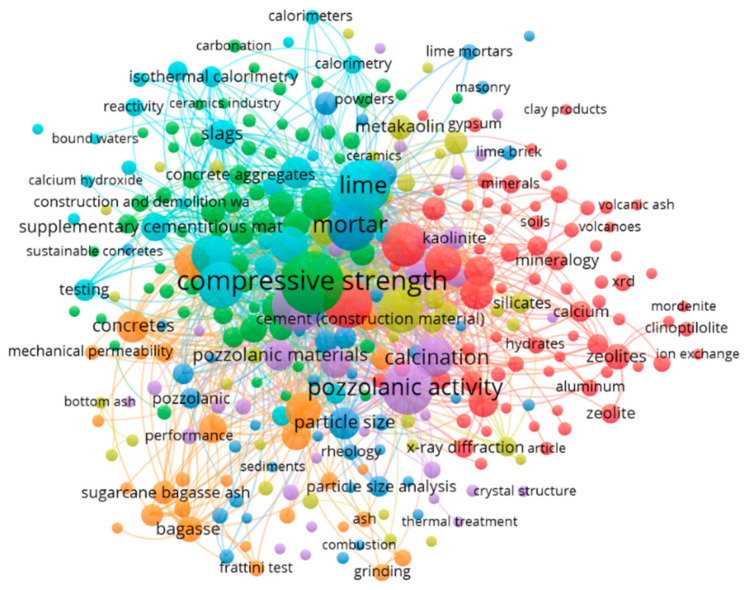
Bibliometric map.

There are a wide range of tests used strictly to evaluate the pozzolanic activity and, among them, analyses are arranged as direct and indirect methods [19,30]. The methods categorized as direct monitor the presence of C-H (Ca(OH)_2_) and its subsequent reduction over time as the pozzolanic reaction proceeds. The pozzolanic reactivity of these methods can be evaluated through the ability of their amorphous phases to react with lime considering the content of reactive silica (SiO_2_) and alumina (Al_2_O_3_) in the material. This process generally causes an environment similar to the hydration of OPC to occur, being a favorable reaction medium between the pozzolanic material and the portlandite. The indirect methods, on the other hand, indicate changes in physical properties revealing the extent of the pozzolanic activity of the material [63]. The tests and methods of analysis of pozzolanic activity can be qualitative when they indicate only if the material has some pozzolanic reactivity or is quantitative when its result is expressed in indices that can be compared with a reference base between more than one sample. For the determination of the pozzolanic activity index, methods are established such as the Frattini test concerning pozzolanicity for pozzolanic OPC [64], modified Chapelle concerning the determination of the fixed C-H content [65], the determination of pozzolanic activity with lime at 7 days [66], the determination of the performance index with OPC at 28 days [67], the determination of pozzolanic activity by electrical conductivity, also called the Luxán method [68], and R^3^ test [69]. In addition to these aforementioned methods, one can take into consideration the tests of isothermal calorimetry, X-ray diffraction, thermal and differential analysis, chemical and mineralogical characterization used to correlate. These methods, along with their presentation and description, are provided in the dedicated section below. Wang et al. [39] point out a greater reliability in the results when these are correlated. As a result, the material should be subjected to a battery of tests if its pozzolanic reactivity is a vehicle for study. Bibliometric research was carried out over the last 10 years, between 2014 and 2023, correlating the keywords: methods, pozzolanicity, clay in the Scopus database and 508 papers were obtained that correlate clay material with pozzolanic reactivity methods. Figure 2 shows the main themes found in the search and among them, it is possible to observe the presence of the Frattini test, thermal analyses such as calorimetry and strength activity index with the compressive strength of mortars. With this, it is possible to observe that one of the most used methods of pozzolanic reactivity analysis is the Frattini test as well as the use of pozzolanic material, in this case clay, in the making of mortars for comparison of their mechanical strength. However, other methods are little considered in research works. One of the objectives of this article is to discuss these methods, evaluating why they are so little used.

### 3.1. Frattini Method

The Frattini test evaluates the consumption of C-H by the pozzolanic reaction of cement and pozzolan [64,70] being established to determine the pozzolanicity of pozzolanic OPC. However, this method is commonly used to gauge the pozzolanic reactivity of some SCM such as calcined clays [71]. The method determines the concentrations of calcium and hydroxide ions dissolved in an aqueous solution containing cement and the pozzolanic material in which its result is obtained from chemical titration. In summary, the concentration of a calcium oxide solution is compared to the sample of pozzolanic material and the quantity needed to saturate a solution with the same hydrogen potential (pH) is calculated. The samples are composed by mixing OPC with the pozzolanic material and then adding 100 mL of freshly prepared deionized water. Then, the samples are placed in a suitable container so that it remains occluded during the curing process determined at a temperature of 40 °C. After curing, the samples should be filtered immediately using a Buchner funnel and cooled to room temperature (RT). The liquid resulting from the filtration process should be analyzed by titration using 0.1 mol/L HCl solution and ethylenediaminetetraacetic acid (EDTA) as indicators of the hydroxyl (OH) and calcium (CaO) phases, respectively. The material is considered pozzolanic when [CaO] and [OH] in solution are below the solubility isotherm used to gauge the results. This method, despite having been idealized and used for a long time to gauge the pozzolanic reactivity, especially for that of cements, is also an analysis used in different SCMs such as brick waste, kaolinitic clays and montmorillonite, natural pozzolans, calcined clays or metakaolin and industrial waste from marble as well as dredging waste and natural ash, for example, which enables comparisons of results in different research and different materials [56,72,73,74]. However, it is important to emphasize that the chemical process of lime fixation by pozzolana is limited by the proper execution of the ideal parameters for the development of hydration reactions, and it is not always possible to establish these criteria in laboratory tests and methods. Due to this, precise and satisfactory results are not always obtained when used with pozzolanic materials such as clays, making Frattini’s method results subject to criticism regarding its accuracy. Among the limiting factors for obtaining accurate results regarding the pozzolanicity of calcined clays, it is possible to highlight that the chemical titration works with very small concentrations and this demands a good execution of the process, besides the preparation of chemical solutions, adequate glassware and utensils, reagents as well as laboratory professionals with mastery in these techniques.

The Frattini test can be performed in a simplified version by the saturated lime method, in which pozzolanic material is combined with a fixed amount of Ca(OH)_2_ in a saturated solution containing lime, OPC and water. Samples should be prepared with the determined amounts of the materials involved in the test in a plastic bottle containing the saturated lime solution. The containers should be kept in an oven at 40 °C for the determined time and soon after, as in the Frattini method, the samples should be filtered and titrated, thus obtaining the amount of fixed CaO. The amount of lime fixed by the pozzolan is determined by the residual measurement of dissolved calcium. This method enables it to be performed at different times without the need for a complete hydration reaction of the OPC [30].

### 3.2. Modified Chapelle Method—Determination of Calcium Hydroxide Fixed (C-H) Content

The determination of the fixed C-H content, known as modified Chapelle, is a method used to assess the reactivity of pozzolanic materials in aqueous media [65,75], based on the ability of the pozzolanic material in addition to fixed lime for the formation of hydrated compounds. This method can be used in several pozzolanic materials; however, many authors consider it an ideal test for calcined clays and metakaolin because it is a process that resembles the hydration of OPC and the consumption of lime with the silica-rich material (pozzolan) [76,77,78]. The modified Chapelle test is standardized and performed in a lime–pozzolan aqueous system for 16 h at 90 °C with continuous stirring. In this process, 2 g of freshly calcined pure virgin lime (CaO) is used in a mixture with 1 g of the pozzolanic material in 250 mL of deionized water at 90 °C for 16 h on average. Concomitantly, a blank test is performed to compare the lime setting index using an equal container with only calcined lime and deionized water. After the thermal treatment, the mixtures should be cooled to RT and stirred in circular movements. The unreacted lime is then extracted by sucrose solution and measured by chemical titration. The result is expressed as the amount of CH consumed by the pozzolanic material. According to Raverdy et al. [79], the minimum C-H consumption result for the material to be classified as pozzolanic is 330 mg CaO/g, a range also described by Aliu et al. [62]. As with Frattini’s test, the Chapelle method is also widely used, mainly in materials complementary to cement, enabling a comparative analysis of pozzolanic reactivity between different materials. As Chapelle’s method presents a similar principle to Frattini’s with the lime setting measured through chemical titration, the method presents congruent limitations and very similar disadvantages of use.

### 3.3. Strength Activity Index (SAI)

The strength activity index (SAI) measures the compressive strength of the blended cement and discriminates the contribution of the pozzolanic reaction to the strength increase [80]. In summary, the SAI is used as a tool to determine a pozzolanic reaction.

Strength development is often used to determine the pozzolanic properties of a material by measuring its reaction with lime and forming cementitious products [81]. The performance index or strength activity is accomplished based on the mechanical compressive strength of mortars with a certain percentage of cement replacement by pozzolanic material as a comparative of a reference mortar to partially evaluate the reactivity according to [46,67]. According to Pormmoon et al. [82], the mortar SAI is measured based on the ratio between the mortar with the pozzolanic material and the reference mortar given in percentage. This method is commonly used since, with the study of SCM, the application of pozzolanic material in partial substitution of cement, is a vehicle to analyze the development of the compressive strength of these cementitious matrices. Due to this, most of the research involving pozzolanic materials tends to instinctively apply this type of analysis. The two most used types of tests involving the strength index or SAI are the methods for determining performance with cement and with lime, in both of which the development of the compressive strength of mortars with aggregate pozzolanic material is used to assess the reactivity of the material. By using mortar compressive strength as an indicator of pozzolanic reactivity, this method presents a great advantage, since this type of investigation links the improvement of a physical property of the cementitious matrices with the pozzolanic activity index of the material. This analysis is one of the most used because, besides already being a practical application of the pozzolanic material, the studies on this theme tend to include the insertion of the material in the scope of the methodology. As such, by applying the material and measuring the pozzolanicity of the pozzolanic material, the cement matrix with the added material can be concomitantly analyzed in different ways. On the other hand, the analysis of pozzolanic reactivity through mortars with partial replacement of cement can be an inconclusive investigation since some fine materials may not present high-pozzolanic reactivity but can indirectly contribute to the improvement in the properties of the cementitious matrix. This happens with the compressive strength and porosity with the filler effect, for example.

### 3.4. Determination of the Performance Index with OPC at 28 Days

This method tends to be confused with the application of the material in mortar for testing. In fact, the pozzolanic performance index or pozzolanic activity index (PAI) with OPC, performed according to the specifications of [67], consists of making two different mortars and their subsequent comparison with respect to their compressive strength. One of the mortars is the reference mortar and the other contains 25% cement replacement by pozzolanic material. In a very similar way, according to Tashima et al. [48], this same method is performed with a lower percentage of pozzolanic material substitution. However, both present the same guideline and objective. In this method, the mortars are made with the appropriate sand and cementitious material ratio as determined by the test methodology as well as with the materials and quantity of specimens that should be molded being determined. The specimens must remain in the curing process for 28 days, and immersion in saturated lime water can be used. After this period, the specimens should be dried in an oven and later subjected to rupture by compression, allowing the calculations of the performance index with Portland cement through the following Equation (1):

(1) $$IAD\;\left(SAI\right)=\frac{\mathrm{Compressive}\;\mathrm{strength}\;\mathrm{of}\;\mathrm{mortar}\;\mathrm{with}\;\mathrm{pozzolanic}\;\mathrm{material}}{Compressive\;strength\;mortar\;reference}$$

### 3.5. Determination of the Pozzolanic Activity Index (PAI) with Lime at 7 Days

The test for determining the PAI with lime is performed with the preparation of mortars, similarly to the PAI/SAI. However, in this method, the prepared mortar must present a part by mass of C-H and another part of pozzolanic material corresponding to twice the volume of C-H. In addition, the mixture must have a pre-established consistency index of 225 ± 5 mm in the fresh state according to normative guidelines [45,66].

The process is identical to the test using OPC, in which specimens are prepared and molded as indicated in the proportions and methodology and then the specimens should be allocated in curing in the molds themselves being kept sealed so that the least loss of moisture occurs over the period of 7 days. The curing must be carried out at RT for 24 h and in an oven at a corresponding temperature of 55 °C (±2 °C) until the seven days are completed. Afterwards the specimens must be cooled down to RT, demolded, capped and broken no later than 4 h after their removal from the oven. For the material to be classified as pozzolan, i.e., to present pozzolanic reactivity, the results should present a compressive strength equal to or greater than 6 MPa.

### 3.6. Determination of Pozzolanic Activity by Electrical Conductivity

Although it is currently considered unusual, due to the existence of more established methods with greater technical and scientific basis, the determination of pozzolanic activity through electrical conductivity is a key analysis for preliminary studies of pozzolanic materials [83,84,85]. This is because this method is considered easily reproducible and fast, and it simply determines whether the material presents some type of pozzolanic activity. However, this method, even considered direct, is not a quantitative analysis since it indicates the presence of an average or good pozzolanicity. The Luxàn method [68] determines the pozzolanic activity through the difference of the electrical conductivity in a period of 120 s of the saturated solution of C-H, aqueous medium vehicle of the reaction and the solution with the added pozzolanic material, making the pozzolanic material to be classified as a material of low, medium or high pozzolanicity [86]. Basically, the method relates the reactivity of the material with the variation in the electrical conductivity of a saturated solution of C-H (Ca(OH)_2_) that the greater the difference of ionic conductivity of the solution after the mixture of Ca(OH)_2_, the greater the confirmation that the material holds pozzolanic reactivity. In the process of performing the test described by Luxàn et al. [68], a saturated solution of C-H is prepared and used as a vehicle for measuring the electrical conductivity as described in the method. To check its reactivity, the material is added to the solution at a temperature of 40 °C for 2 min, but the electrical conductivity value must first be measured. Once the determined time has elapsed, the electrical conductivity value is measured again and the initial and final values are subtracted to obtain the test results according to Equation (2) and qualifying them in materials that do not present pozzolanic reactivity when the potential difference is less than 0.4 mS/cm, variable or medium pozzolanicity with results between 0.4 and 1.20 mS/cm, and good pozzolanicity with values above 1.20 mS/cm [68].

(2) $${\Delta C}_{40{}^{\circ}}=C-C_{0}$$

where: ΔC_40_: electrical conductivity at 40 °C; C_0_: initial electrical conductivity at 40 °C without pozzolanic material; C: final electrical conductivity at 40 °C with pozzolanic material.

However, as a limitation, this method is rarely used and this makes it impossible to have comparability parameters with other studies about pozzolans, especially in the case of calcined clays. This is due to its limited accuracy in quantifying the pozzolanic reactivity of the material as well as the possibility that other factors increase its electrical conductivity that may not be related to the pozzolanic reaction. As a result, this method is considered only qualitative and has three classifications regarding reactivity. Even so, this test has a high reproducibility and a quick response, thus optimizing preliminary tests.

### 3.7. R^3^ Test—Rapid, Relevant and Reliable

Avet et al. [69,87] point out that there are numerous tests for determining the pozzolanic activity of SCM; however, not all correlate well with the compressive strength of mortars and concretes with added pozzolans.

Different from the other methods, the R^3^ test was devised with the purpose of analyzing the pozzolanic activity of a given type of material. In this case, Avet et al. [69,87], the developer of the method, uses this analysis for pozzolanic activity tests especially in calcined clays. The R^3^ test is based on the correlation between the chemical reactivity of calcined clays in a simplified system using only a muffle furnace and an oven. In summary, the R3 test uses a condensed system to separately measure the reaction of a pozzolanic material with less interference and without accounting for other reactions in the clinker hydration. In the realization of this R^3^ method, the bound water is determined with the aid of thermogravimetry performed with simple elements. This analysis uses a paste of pozzolanic material and portlandite in a 2:1 mixture of pozzolan and lime and a water/cement ratio of 1.2 with the pH of the solution adjusted in the 13.3 to 13.7 ranges. The samples are placed in hermetically sealed containers in an oven at 40 °C for 24 h and then the material is removed and demolded from the container and cut into three slices approximately 4 mm thick using a diamond saw. The slices are dried in a 110 °C oven with permanence until the mass variation does not exceed 0.5%, approximately 48 h. After this period, the mass value of each sample will be weighed and identified. Then they will be subjected to a temperature of 400 °C in a muffle furnace for 2 h. After the heating process, the samples will be cooled down to 110 °C and weighed again. The bound water will be determined according to Equation (3).

(3) $${\%\;}_{Bound\;water}=\frac{m110\;{}^{\circ}C-m\;400-110\;{}^{\circ}C}{\;m110\;{}^{\circ}C}$$

where % bound water = percent water bound of the material; m110 °C = sample mass in an oven stabilized at 110 °C; m400—110 °C = mass of the sample heated to 400 °C and cooled to 110 °C. In addition to determining the pozzolanicity index by water bonding using furnace heat treatment, heat flow measurements using isothermal calorimetry are also a correlation to gauge the reactivity of the material based on cumulative heat release and X-ray diffraction. In this correlation method, the cumulative heat of the reaction is measured in an isothermal calorimeter for 7 days at 20 °C and 40 °C. The paste is constructed and stored, including the mixing solutions, which must be stored at the same temperature as the test and then mixed for testing in the calorimeter. With that, the samples are made and melted to characterize the crystalline phases by means of XRD as described in the procedure.

This R^3^ method is relatively new and was created mainly to assess the pozzolanic reactivity of calcined clays. A major advantage of its use is the correlation that this test makes with other methods and with basic and fundamental concepts such as the quantification of water bound in the material. This method also demands less time for the results to be obtained as well as simpler equipment and methods to be used.

## 4. Analysis of the Approaches for Pozzolanic Activity Characterization: Potentialities and Limitations

Besides the methods used to measure the pozzolanic reactivity of SCM, especially calcined clays, there are commonly employed analyses to characterize materials, which provide an indication of pozzolanic reactivity. The utilization of these methods, in conjunction with pozzolanic activity analyses, tends to provide a more robust confirmation of the material’s reactivity.

As the pozzolanic behavior of the materials presents important changes in different properties, it is necessary to perform a more extensive analysis to determine the origin of the variations. In general, the most commonly used investigations as complementary tests for pozzolanic activity are the specific surface area analysis, XRD, X-ray fluorescence spectrometry (XRF) and thermal analysis.

The analysis of the specific surface area is an important factor in the quality control of pulverulent materials, which in general are the SCMs. The determination of the specific surface area of a material directly correlates its fineness with the physical properties that the material can provide with its use, in addition to particle size, amount of pores, material uniformity and surface energy. This method is suitable for determining the surface area of high-fineness materials, which is very important in studies of clays, for example, since the behavior of this material can be distinct at different levels of fineness. The degree of fineness of the material is directly related to the calcination processes, water demand, workability and reactivity [22,88].

XRD is a widely used resource in research to identify and quantify the mineral phases present in a sample. For analysis of clay materials, especially calcined clays, quantitative analysis has wide use [88]. This investigation analyzes the argillominerals by pointing out the minerals present in the analyzed materials, identifying the crystalline as mullite, quartz, hematite and kaolinite and amorphous phases as metakaolinite, silica, aluminates and oxides present.

This type of test is performed in diffractometer-type equipment that can operate with Cu-Kα radiation, for example, and present sweeps of angular bands with wide variations and usually a diffraction angle of 2θ. The speed of the test is also variable, and this is strictly related to the specific detail in the results. For identification and quantification of the crystalline phases, refinement methods are used with computer software databases. This step relies on the analysis of chemical composition, selectivity and preferential direction, structural disorder and structural differences between clay minerals [89,90].

XRD is usually used to differentiate materials that have the same clay minerals but with different origin in order to validate the purification process of these materials [91]. Many phases of clay materials such as chlorite minerals, cristobalite, halloysite and quartz, for example, can only be identified through XRD [92]. In addition, this technique is widely used to identify the compounds resulting from mixtures of materials in order to link the enhancement of properties such as pozzolanic reactivity, lower water requirement and delayed hydration as well as mechanical strength with the development of new phases and compounds [7]. With this, it is possible to point to the XRD as one of the most effective techniques for the characterization of clay minerals.

XRF is a non-destructive method used to perform the elemental analysis, also known as chemical analysis, of samples. This technique is based on photoelectricity in which the atoms are excited with an energy source capable of removing electrons from internal orbitals, generating a release of space that will be filled by electrons from more external orbitals. This gap filling generates characteristic X-ray emission, with distinct energies, and ultimately indicates which element has a particular energy dispersion.

In XRF, elemental analysis is performed with pulverized raw material in a spectrometer and its results are obtained by a base acquisition in which it is possible to identify the primary elements of the material. In the case of clay materials, elemental analysis, also known as chemical analysis, is a complementary method to XRD considering that the primary and elemental elements of the sample are obtained and the crystalline phases are detected, which in general occur from already formed compounds. Without elemental analysis, the identification of the compounds of the material is difficult and often incorrect and incomplete.

The elemental or chemical analysis of materials is paramount in the characterization of their content. This investigation aims to outline strategies for applications and the processing of the material according to the elements arranged [93]. Thus, this method presents great usability in the study of SCM with the goals of gauging the potential replacement or complementation of clinker in the elements present in the material [94].

As a result, it is possible to observe that the elemental analysis of materials is an important factor to guide investigations in different areas. However, its use is strongly allied to the characterization of soils and especially clayey materials. Due to this, besides the study of materials complementary to OPC, XRF can be applied in the measurement of toxic elements in soils [95]. The content of relevant elements in soil such as cadmium [96] is important for mixed historical pigments [97] and in the analysis of elements in acid-activated clays used to improve the quality of oils [98] for example.

In addition to the methods of characterization for materials related to their formation, investigation of their elements, and chemical and mineralogical research, there are methods and thermal analysis that show changes in the behavior of materials as a result of temperature changes. It is worth mentioning that even with several tests available to thermally analyze the material, not all methods are suitable for clay materials. Table 2 presents a summary of methods that can be used to investigate clays in general, depending on their use and the purpose of the analysis. However, the most commonly used methods for clays, whether calcined or crude, are TGA, DTG, DTA and DSC.

Differential thermal analysis (DTA) and thermogravimetric analysis (TGA) are widely used to determine the thermal behavior of clays. The TGA technique follows the variation of the sample mass, as a function of temperature with programming variation. The DTG is a mathematical arrangement that is the first derivative of TGA as a function of time or temperature. TGA is a tool used for the purpose of gauging the C-H content within a cementitious system. While the isothermal calorimeter points out both the heat evolution and the cumulative heat generation in a cementitious system. They are considered important tools in studying the pozzolanic behavior of SCM [24,99].

Another commonly used method is differential exploratory calorimetry (DSC) which covers research on new cements and SCM. This thermoanalytical method monitors enthalpy reactions against a thermally inert reference material while both are subjected to a programmed and controlled temperature ramp. Basic isothermal calorimetry is used to evaluate the hydration of pastes produced with the clays.

Thermal analyses, especially thermogravimetry and calorimetry, are basically used to investigate the behavior of the clays when subjected to temperature elevation and in the hydration process, by investigating the release and flow of heat from the mixtures for phase identification. These types of tests serve to demonstrate both the behavior of cement hydration when clays are added and the change in mass during the temperature change [100,101,102,103,104,105,106].

Identification methods of clay minerals are crucial methods to better understand the material and to envision a viable application to these compounds. Due to the high degree of fineness of the particle size of clay materials and the electrical charges they possess, the identification, recognition, understanding and classification of clay minerals tends to present a higher degree of complexity as these materials exhibit a dynamic and unpredictable behavior. Due to its behavior, the application of this material requires deep studies since it has the ability to exert positive or negative influences with its use [107].

Table 3 shows selected studies from 2014 to 2023 that present investigations about the reactivity of calcined clays or similar materials in different applications as well as their main results.

This table shows works that investigated pozzolanic clay materials as well as their applications as a substitute or complementary material for OPC. Articles from high-impact journals were chosen and selected, focusing on research involving calcined clays in which the results encompassed the pozzolanic activity of the material. It is possible to highlight that for a better understanding and a more complete analysis of the pozzolanic materials, especially the clay-based materials, it is necessary to observe the results of different evaluation methods that directly or indirectly evaluate the pozzolanic activity. In most studies, the use of pozzolanicity analysis methods in conjunction with another test that is usually used to characterize the material is performed.

**Figure 3:**
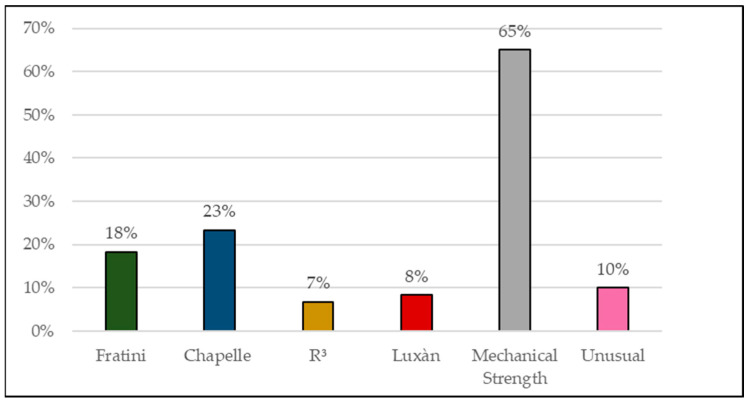
Percentage of use of Pozzolanic reactivity tests.

**Figure 4:**
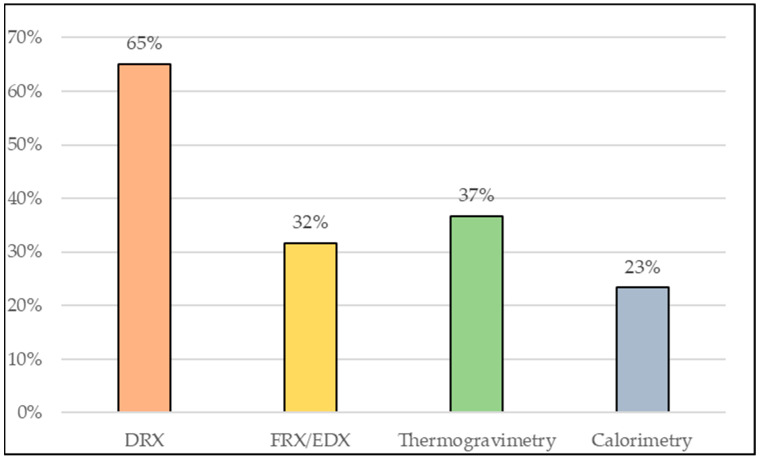
Percentage use of characterization tests. Among the methods, the most used are those classified as direct, which measure changes in material properties. The graph in Figure 5 shows that only 5% of the studies did not apply any method, and this was due to the use of materials considered already established as pozzolanic in the literature or that have been previously studied. However, 25% of the cases used direct and indirect tests together.

The use of multiple methods to evaluate the pozzolanic reactivity of clay materials is a result of the need for a better understanding of the origin of their potentialization. It is also important to evaluate research on the properties of cement matrices with pozzolanic materials of clay origin, since the improvement of properties is not always derived from factors linked only to pozzolanic reactivity. For this reason, research such as that of Yanguatin et al. [126] and Tole et al. [114] have used various methods for a more in-depth analysis of the materials. Figure 3 shows a graph with indicative percentages of the use of the aforementioned tests based on the research catalogued in Table 3. It can be seen that the investigation of mechanical strength is the most used analysis to assess the pozzolanic reactivity of these materials when the objective is the partial replacement of OPC, followed by the Chapelle and Frattini methods. As for the characterization tests, it can be stated based on the graph in Figure 4 that XRD is the most used test and this is due to the importance of the mineralogical characterization of the material.

## 5. Conclusions

In view of the analysis of the methods related to the pozzolanic reactivity of supplementary cementitious materials (SCMs), especially in calcined clays, it can be observed that the tests, in general, need to be associated with more reliable results [76]. Pozzolanic potential analyses involve the creation of a medium that resembles, at least in part, the cement hydration reaction, either in temperature, pH or time, causing the calcium hydroxide (C-H) to react with the pozzolanic material. In these tests, the C-H can be added to a favorable environment for the reaction to take place, as in the formation of a saturated solution, or in a more applicable way, obtaining the cement portlandite.

It is worth mentioning that some tests, such as the determination of the performance index, whether with lime or cement, do not present a high degree of reliability of pozzolanic reactivity, since the increase in mortar resistance may be related to other factors such as the filling effect and filler. It is possible to highlight that to correctly assess the improvement in the characteristics of a cementitious matrix, it is necessary to cross-reference direct, indirect and material characterization tests.

However, some of these methods are extremely complex to be reproduced in any laboratory, as well as their performance time, which is very long, making the most complete research of these materials longer. However, tests such as that proposed by Lùxan et al. [68], which are simpler, might be considered unreliable because their use is currently very low, although its operating principle is well correlated to widely used tests such as Chapelle and Frattini. Throughout the work, it is possible to observe that for the study of clays, it is very important to characterize and know the elements of the material. X-ray diffraction (XRD) plays a pivotal role in the investigation of calcined clays. This technique provides valuable insights into the crystalline structure and mineralogical composition, thereby enhancing understanding of the physical properties of the materials. Consequently, XRD is frequently employed in tandem with pozzolanicity index tests to validate the material’s reactivity. In the case of calcined clays, the stages of transformation and the material’s behavior when subjected to temperature changes were analyzed. In the pozzolanic materials, it is possible to observe a different behavior from the materials that do not present reactivity as well as a difference in the cement hydration products when subjected to calorimetry, for example.

By observing the methods for analyzing pozzolanic materials and their procedures, it is possible to highlight that even with different procedures, there is an approximation regarding the main theory of conceiving a medium and an environment, similar to the cement hydration, that is favorable to the reaction of the pozzolanic material with C-H. Despite the complexity of the analysis of the SCM and the use of clay as a substitute for cement, since, as presented throughout the work, there is no consensus about the calcination temperature, the amount of clay minerals and the effectiveness of the methods of evaluation of the pozzolanic reactivity of this material. However, one can highlight that the calcined clays tend to ensure important properties to cementitious matrices with a lower CO_2_ emission and, depending on the way of processing, a lower final cost. In summary, it is possible to highlight that an accentuated and complete analysis in the area of SCM requires both tests to determine the pozzolanic activity or reactivity of the material as well as an investigation with methods that assess their primary characteristics such as thermal behavior and characterization tests. In general, the tests to determine the pozzolanic activity index indicate possible behavior of the material, creating the need to observe its characteristics by different means. In the course of the work, it was possible to realize that the most used analyses in recent studies regarding calcined clays are the Frattini method, analysis of the mechanical resistance of mortars with the incorporation of pozzolanic material, determination of the fixed C-H content, also known as modified Chapelle and, together, XRD. These two techniques are well regarded for their uses in referential research in the area of SCM and for presenting qualitative and quantitative results with a high degree of reliability.

## Figures and Tables

**Figure 5:**
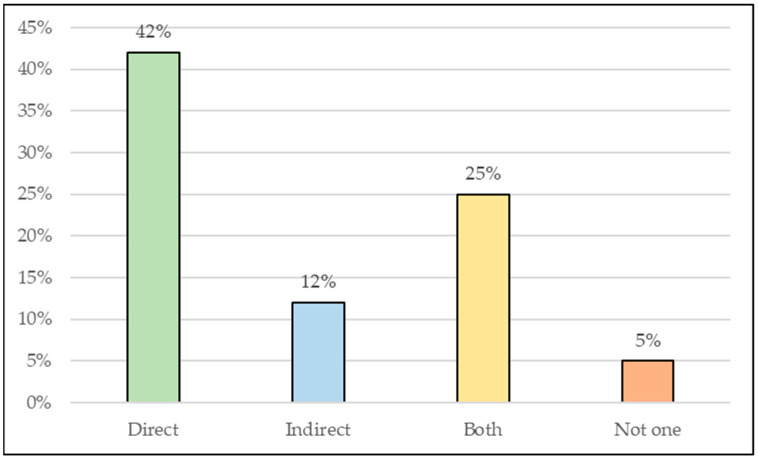
Percentage use of tests according to classification.

**Table 1:** Supplementary and complementary characteristics between OPC and SCM.

Material Characteristics	OPC	SMC
Composition/source	Produced from raw materials such as limestone, clay and, to a lesser extent, minerals.	Coming from by-products or residues from industrial processes, such as coal fly ash, blast furnace slag, and silica fume, natural materials or those submitted to activation processes.
Contribution to mechanical strength	Mainly responsible for the compressive strength of cementitious matrices, with a solid and cohesive formation due to the hydration process.	They can contribute to concrete strength. Materials such as fly ash and blast furnace slag that have pozzolanic reactivity react with the calcium hydroxide released during cement hydration forming additional compounds and strengthening the concrete matrix.
Influence on durability	It provides good durability in ordinary environments; however, it has high alkalinity that can be unfavorable in aggressive environmental conditions.	They can show significant improvement in the durability of cementitious matrices in aggressive environments by increasing resistance to chloride ion penetration and enhance corrosion protection.
Sustainability	High energy and CO_2_ emission during clinker production.	Used to reduce Portland cement consumption, which results in less environmental impact.
Hardening time	Intense and fast hydration process.	They make it possible to delay the hardening time, seen as an advantage in certain applications.
Shrinkage	Tendency to shrink during the hardening process.	Helps reduce concrete shrinkage, due to its lower amount of hydration compounds.
Fineness and influence on reactivity	Directly related to its reactivity and hydration rate. The thinner the cement results in a faster hydration rate.	In general, finer supplementary materials have a greater pozzolanic reactivity, because they have a larger surface area available to react with the calcium hydroxide released during cement hydration. These materials can also interfere with the physical characteristics of the cementitious matrix, since they can present a filling effect.

**Table 2:** Thermal analysis results.

Technical	Abbreviature	Property	Use
Thermogravimetric analysis	TGA	Mass	Decomposition
Derivative thermogravimetry	DTG	Mass	Dehydration, oxidation
Differential thermal analysis	DTA	Temperature	Phase change, reactions
Differential scanning calorimetry	DSC	Enthalpy	Heat capacity, phase change reactions, calorimetry
Thermomechanical analysis	TMA	Deformation	Mechanical change expansion
Thermoptometry	-	Optics	Phase change Surface reactions coloration Changes

**Table 3:** Research and tests used to assess the pozzolanic activity of clays.

Research		Methods Employed	Main Results
01	[108]	Thermogravimetric analysis, calorimetry, mechanical strength and X-ray diffraction	Increased compressive strength verified by strength activity index test; observation in XRD diffraction characterization of a higher cement substitution factor and slow pozzolanic reaction, progress of pozzolanic reaction observed with thermal analysis.
02	[109]	Mechanical strength	Progression of compressive strength at all ages verified by strength activity index test.
03	[110]	Chapelle, thermogravimetric analyses, X-ray diffraction and X-ray fluorescence	It was observed with the compressive strength that the materials compared to the clay show distinct performance; TGA pointed out total portlandite was consumed representing good pozzolanic reaction of the calcined clay.
04	[111]	Mechanical strength and X-ray diffraction	XRD was used to characterize the clay to form the metakaolin and mechanical strength to measure the progress of the compressive strength of the geopolymer.
05	[112]	Mechanical strength	Observation of the increase or similarity of the mechanical strength of concretes with calcined clay.
06	[113]	Mechanical strength	Good behavior of metakaolin with steel fibers and increased compressive strength, especially at older ages.
07	[42]	Chapelle, mechanical resistance and X-ray diffraction	XRD was used to characterize the clays, observing the complete dehydroxylation of kaolinite after calcination at 750 °C, pointing to pozzolanic reactivity through the chaplet method, confirmed by thermal analysis, and the compressive strengths of the mortars were equal or superior to the reference ones.
08	[114]	Frattini, mechanical resistance, X-ray diffraction and X-ray fluorescence	Mechanochemical activation potentiated the pozzolanic activity of natural clays, the strength activity index, and the Frattini test confirmed the increase in pozzolanic reactivity; XRD investigations pointed out the ability of clays to act as pozzolans.
09	[115]	Mechanical strength and X-ray diffraction	XDR pointed out the dehydroxylation of kaolinite at 600 °C and the strength activity index showed higher compressive strengths at later ages.
10	[116]	Calorimetry and X-ray diffraction	XRD was used for characterization and gauged the presence of kaolinite in the clays, isothermal calorimetry was used to rapidly probe the reactivity of kaolinite clays, and thermal analysis quantified the kaolinite showing a strict directly proportional link between the kaolinite content of the crude clay and reactivity of the calcined clay.
11	[19]	R^3^, X-ray diffraction and X-ray fluorescence	Calcined clays were characterized by XRD and XFR, pointing out the potentiality of the material and the pozzolanic R^3^ test showed that the clays are significantly more reactive than fly ash and comparable to slag and silica fume.
12	[117]	Frattini, Luxàn, X-ray diffraction, X-ray fluorescence, thermogravimetric analysis and mechanical strength	The XRD indicated the potentiality of the material in its characterization, the electrical conductivity confirmed that the natural pozzolans can be classified as of low reactivity, the Frattini test and the thermogravimetric analysis confirmed the pozzolanic behavior of the natural pozzolans being these results confirmed by mechanical tests with strength gain around 13 to 15% at advanced ages.
13	[118]	R^3^, X-ray diffraction and X-ray fluorescence	XRD was possible the mineralogical characterization of 13 different clay-rich raw materials pointing to an evaluation of the previous pozzolanic reactivity being confirmed by the R^3^ calorimetry test where kaolinite influences the heat of hydration.
14	[119]	Chapelle, R^3^, X-ray diffraction and mechanical strength	The XRD was a primary analysis to explain the difference in behavior between the materials; there was an increase in the strength of the mortars with calcined clay measured by compressive strength, the R^3^ test also showed pozzolanic reactivity of the clays.
15	[120]	Chapelle and X-ray diffraction	The XRF characterized and pointed out the pozzolanic activity of the material as well as the XRD revealed that the presence of the material in the system increases the presence of CSH. The Chapelle test confirmed that the increase in compressive strength was due to the pozzolanic reaction of the material.
16	[7]	Unusual comparative analysis, X-ray diffraction and X-ray fluorescence	The XRF and XRD characterized the material pointing pozzolanic potential. The pozzolanic reactivity was measured by unconventional tests.
17	[76]	Chapelle, thermogravimetric analysis, X-ray diffraction and X-ray fluorescence	The XRF and XRD characterized the material, pointing to pozzolanic potential. The combination of X-ray diffraction (XRD) and thermogravimetric (TG) techniques differentiated the difference of calcining and grinding. The Chapelle test pointed out better pozzolanic reactivity of the material compared to the commercial one.
18	[105]	Mechanical strength	The mechanical strength was used to assess the pozzolanic development of the material in the cement matrix.
19	[121]	Mechanical resistance, calorimetry and X-ray fluorescence	The XRD was used to measure the crystalline phases. The calorimetry showed the formation of hydrates pointing to a pozzolanic reaction with the formation of CSH. The compressive strength of the cementitious matrices showed a decrease when percentages higher than 30% were substituted as well as presenting the development of pozzolanic reactivity of the material.
20	[122]	Mechanical strength, Luxàn and X-ray fluorescence	XRF was used for characterization of the materials. The Lùxan test indicated a medium pozzolanicity of the material that induces filling behavior in the cement matrix. The incorporation of the residue increased the mechanical strength.
21	[57]	Thermogravimetric analysis, calorimetry and X-ray diffraction	The results of calorimetry, XRD and TG revealed the pozzolanic reaction of the calcined clay, and due to the pozzolanic reaction, there was a greater production of HSC increasing the mechanical strength.
22	[123]	Mechanical strength, unusual comparative analyses, X-ray diffraction and calorimetry	XRD and DTA/TG studies confirmed declines in portlandite content and CSH formation. Using less usual standardized tests, the cements were considered pozzolanic. Mechanical performance analysis reinforced the pozzolanic potentiality of the material.
23	[124]	Mechanical strength, unusual comparative analyses and thermogravimetric analysis	The aforementioned tests have shown that the red clay has a higher potential compared to the others.
24	[125]	Mechanical strength	The pozzolanic potential of the materials was tested by the mechanical behavior of mortars, showing a positive effect on the properties. It was observed that the pozzolanic materials can improve the environmental resistance of the mortar.
25	[126]	Frattini, Chapelle, mechanical strength, thermogravimetric analysis, calorimetry, X-ray diffraction and X-ray fluorescence	The pozzolanic activity of the clays was evaluated by calcium hydroxide fixation (Chapelle), Frattini test and compressive strength, indicating that when calcined at 650 °C, they presented pozzolanic activity and a percentage increase in compressive strength.
26	[127]	Chapelle, mechanical strength	The results of the pozzolanic activity evaluation by the Chapelle method showed that this material has potential to be used as a pozzolan. In the mechanical strength analysis, the ternary mixtures with the material and limestone resulted in higher compressive strength.
27	[128]	Mechanical strength, thermogravimetric analysis, X-ray diffraction and X-ray fluorescence	The thermal transformation of the materials was studied by TG-DTA and XRD, pointing out the temperatures of the transformations of each phase. The pozzolanicity was measured at 7 days and the strength activity index by mechanical resistance had a significant increase. The material, when calcined, presents itself as a reactive pozzolan but with later reactions.
28	[129]	Chapelle and mechanical strength	The Chapelle test and the resistance activity index were used to evaluate the pozzolanic activity of the material after thermal treatment, indicating higher pozzolanic activity when calcined.
29	[86]	Luxán	The evaluation of pozzolanicity was necessary to understand how the material behaves in the reactive process with the cement used in the matrix and showed good pozzolanic reactivity according to the Lùxan method, enabling improvements in technological properties such as mechanical strength.
30	[130]	Frattini, mechanical strength, X-ray diffraction and X-ray fluorescence	With the characterization tests, such as XRD, mechanical resistance analysis and pozzolanicity tests, it was possible to determine that the clays can replace cement in mortars. The incorporation of the material increased the density of the mortar; however, its mechanical resistance decreased.
31	[131]	Mechanical strength, unusual comparative analyses, X-ray diffraction and X-ray fluorescence	With the applied methodology, it was possible to observe that the partial replacement of cement in the mortars by clays resulted in considerable improvement in the compressive strength at 28 days. The pozzolanic reactivity of clays A and B is explained by the characterization of structural changes after calcination with XRD, thermal and unconventional analysis.
32	[99]	Mechanical resistance, thermogravimetric analysis and calorimetry	The resistance activity indexes meet the requirements for a pozzolanic material. The results of compressive strength indicated that the optimum strength to replace Portland cement up to 30% was viable for the use of the brick waste.
33	[132]	Frattini	With Frattini’s method it was possible to evaluate that the material in question ci exhibited greater pozzolanic activity than non-activated blended cement samples, even though it was considered non-pozzolanic.
34	[133]	Unusual comparative analysis and X-ray diffraction	During the work, the characterization of the material was carried out by XRD and unconventional methods to assess the development of pozzolanic reactivity. The calcium adsorption by kaolinite increased with the increase in the initial concentration of hydrated lime causing the pozzolanic reaction to be delayed in the long term.
35	[134]	Mechanical strength and X-ray diffraction	To assess the pozzolanic activity, XRD was used for material characterization and mechanical resynthesis to assess the pozzolanic development. The results showed a decline in the physical properties of the material as well as in the mechanical strength when larger size material is used.
36	[135]	Chapelle, thermogravimetric analysis and X-ray diffraction	The pozzolanic potentials were investigated using a novel method involving TGA/dTG and XRD techniques, in addition to the Chapelle method. The results provided insights for the development of restoration mortars.
37	[136]	Thermogravimetric analysis and X-ray diffraction	The mineralogical changes were monitored through X-ray diffraction (XRD) and thermogravimetric analysis. Different lime contents of the treated samples were used to assess the development of pozzolanic reactions. It was observed that the clays exhibited low reactivity, resulting in a delay in the precipitation of new hydrated phases.
38	[137]	Frattini, mechanical strength and Luxàn	The materials, 3 clays, were selected and characterized. After calcination at 700 °C and grinding, the pozzolanic activity was determined by means of the electrical conductivity test, Frattini test and compressive strength index. The results show that all calcined clays are classified as highly reactive pozzolanic.
39	[138]	Mechanical strength, thermogravimetric analysis, calorimetry, X-ray diffraction and X-ray fluorescence	Preliminary analyses such as XRD show a difference in its metakaolinite content. Isothermal calorimetry and compressive strength tests were performed, complementing the analysis of the development of pozzolanic reactivity in the matrix being monitored the hydration reaction by thermogravimetric analysis (TGA). As a main result, it was found that the reactivity of the materials are distinct.
40	[139]	Frattini, mechanical strength, calorimetry, X-ray diffraction and X-ray fluorescence	In this work, it was observed that the clay can be calcined to form a technically feasible supplementary cementitious material since the compressive strength of the concrete showed results similar to other materials already in use. The XRD characterization showed a reduction in crystallinity and increase in amorphous phases in samples calcined at 600 ºC confirmed by thermal analyses. The Frattini test indicates continuous pozzolanic reaction with curing time.
41	[140]	Mechanical strength	The incorporation and additions in the cement allowed for mortars to be obtained with higher physical-mechanical properties and durability than the reference mortar. However, mixtures containing glass powder and/or metakaolin showed higher strength than those containing brick waste.
42	[141]	Chapelle and X-ray diffraction	XRD was performed to characterize and quantify the amount of silica in the material. With the modified Chapelle test, it was possible to determine that the highest amounts of fixed lime were found at 700 and 800 °C, i.e., the highest pozzolanic reactivity at these temperatures.
43	[102]	Frattini and X-ray fluorescence	In conjunction with XRF and Frattini’s method it was observed that at higher temperatures a decrease in reactivity occurs. The reactivity of the clay increases directly proportional to the calcination temperature, with the limit at a temperature of 900 °C.
44	[69]	Chapelle, mechanical strength, R^3^ and calorimetry	Very good correlations were found between the compressive strength of the mortar and both measurements of chemical reactivity, presenting a new and fast method indicated especially for calcined clays, first being obtained by measuring the heat release during the reaction using isothermal calorimetry and second by determining the bound water.
45	[142]	Mechanical strength, thermogravimetric analysis and calorimetry	According to the mechanical strength, thermogravimetric analysis and calorimetry methods, the ideal heat treatment is 700 °C, the replacement rate of clinker for the material is 10% and the mortars containing the material have good mechanical strength.
46	[143]	Mechanical strength	The partial replacement of ordinary Portland cement by calcined clay in the mortars showed equal strength and decreased thermal conductivities.
47	[144]	Mechanical strength, thermogravimetric and calorimetric analysis and X-ray diffraction	Thermogravimetric, differential scanning calorimetry and X-ray diffraction analyses were performed to find out the evolution of the microstructure of the mortar with the material as well as analysis of its development with respect to compressive strength. With this, it can be concluded that the material improved the acid attack resistance in the mortars, increasing the CSH content, resulting in filler and pozzolanic activity.
48	[145]	Mechanical strength, thermogravimetric analysis and X-ray diffraction and X-ray fluorescence	In this work, the results obtained from the analysis of the material indicated its potential use in mortars when calcined at 750, 850, and 950 °C. For this, physical, chemical, mechanical and microstructural analyses were performed. The XRD revealed the dehydroxylation and transformation of kaolinite into metakaolin, confirmed by thermal analysis. The mechanical performance of all mortars increases steadily with age.
49	[4]	Mechanical strength, thermogravimetric analysis and calorimetry and X-ray diffraction and X-ray fluorescence	Reactive pozzolans can be obtained from low-grade kaolinitic clays, if properly calcined. The material was characterized by XRD, XRF and TGA-DTA. The pozzolanic reactivity was evaluated by compressive strength in mortars. The evolution of pozzolanic reactivity is limited at temperatures between 800 and 900 °C. The results point out that clays with moderate kaolinite contents constitute a potential source of high reactivity for pozzolanic materials.
50	[146]	Chapelle, thermogravimetric analysis and X-ray diffraction	The calcined materials at temperatures of 600–900 °C were investigated by XRD and TGA techniques and the Chapelle test. The presence of alumina and its subsequent dehydroxylation in the calcination process was observed and confirmed by thermal analysis. The Chapelle test indicates a better pozzolanic reactivity at 800 º C where the density of the hydrates increased. The calcination of the material is strictly connected with its microstructure and pozzolanic activity.
51	[147]	Frattini and mechanical strength	The pozzolanic activity of the material was gauged by the Frattini test in conjunction with the compressive strength. The results show that the replacement in a percentage of 30% presents an improvement in the mechanical behavior.
52	[148]	Unusual comparative analysis, thermogravimetric Analysis, X-ray Diffraction and X-ray Fluorescence	The investigation of the materials was carried out at temperatures of 600–850 °C for the production of metakaolin with pozzolanic activity with the DTA/TG and XRD methods in order to specify the mineralogical composition and the extent of dehydroxylation. The pozzolanic activity was evaluated in order to gauge the optimum calcination temperature, obtained at 700 °C and 800 °C. The pozzolanic activity content for the materials was distinct at about 20%.
53	[149]	Chapelle, mechanical strength, thermogravimetric analysis and X-ray diffraction	Analyzing the results, it was possible to observe that the optimum condition for calcination to be 800 °C, presenting total dihydroxylation and greater pozzolanic reactivity. The research points out a percentage of 15% for ideal content of metakaolin produced based on the compressive strength since the mechanical properties were superior.
54	[150]	Chapelle, mechanical strength, thermogravimetric analysis and X-ray diffraction	In this work, the Chapelle test was used as screening to assess the potential materials to be used in mortars. The mechanical strength of the cementitious matrices was improved when compared to the use of other materials in OPC incorporation.
55	[151]	Mechanical strength and X-ray diffraction	In this work, the characterization of the material was carried out, and despite being crystalline, it presented pozzolanic activity and this can be verified by the methods of analysis in the work. Furthermore, the compressive strength of mortars was improved by the calcination process at high temperatures.
56	[152]	Frattini and mechanical strength	The results present both calcined kaolinitic clays as pozzolanic and effective with different reaction rates that is directly related to their specific surface area in the post-calcination process besides their crystalline structure. The material presents a very high-pozzolanic activity allowing high levels of substitution.
57	[153]	Mechanical strength, thermogravimetric analysis, calorimetry, X-ray diffraction and X-ray fluorescence	The investigation provided the increase in reactivity of calcined clays by different factors. With the thermal treatment and with the analysis methods, an increase in the pozzolanic reactivity of the SCM was observed, which resulted in higher compressive strengths.
58	[154]	Mechanical strength, Luxán, thermogravimetric analysis and X-ray diffraction	The experimental results indicated that the calcined material has a higher strength performance if compared to its natural state and this is due to the increased pozzolanic reactivity as well as the decreased porosity and refined pore structure of the hardened cementitious system.
59	[155]	Mechanical strength and X-ray diffraction	This work assessed the technical feasibility of introducing up to 20% by weight of red mud in mortars. The gain in compressive strength was not attributed to the pozzolanic reaction because in its characterization, the material showed no evidence of pozzolanic reaction.
60	[156]	Frattini, mechanical strength, thermogravimetric analysis and X-ray diffraction	The five clays were characterized by XRD, ATG and DTA chemical analysis and pointed out pozzolanic reactivity of the materials. However, the pozzolanic activity of the clays depends on the kaolinite content and their crystalline structure in which clays containing more than 50% kaolinite produce more reactive material. In the results, it is possible to observe the presence of alginate pozzolanic reactivity evaluated by the Frattini test and increased compressive strength.

## Data Availability

Not applicable.
